# LC-QTOF-MS analysis of xanthone content in different parts of *Garcinia mangostana* and its influence on cholinesterase inhibition

**DOI:** 10.1080/14756366.2020.1786819

**Published:** 2020-07-01

**Authors:** Kooi Yeong Khaw, Chun Wie Chong, Vikneswaran Murugaiyah

**Affiliations:** a School of Pharmacy, Monash University Malaysia, Jalan Lagoon Selatan, Bandar Sunway, Subang Jaya, Selangor, Malaysia; b Discipline of Pharmacology, School of Pharmaceutical Sciences, Universiti Sains Malaysia, Penang, Malaysia

**Keywords:** α-Mangostin, γ-mangostin, *Garcinia mangostana*, LC-QTOF-MS, xanthone, cholinesterase inhibition

## Abstract

Mangosteen is one of the best tasting tropical fruit widely cultivated in Southeast Asia. This study aimed to quantify xanthone content in different parts of *Garcinia mangostana* by LC-QTOF-MS and determine its influence on their cholinesterase inhibitory activities. The total xanthone content in *G. mangostana* was in the following order: pericarp > calyx > bark > stalk > stem > leaves > aril. The total xanthone content of pericarp was 100 times higher than the aril. Methanol extracts of the pericarp and calyx demonstrated the most potent inhibitory activities against acetylcholinesterase (AChE) and butyrylcholinesterase (BChE) with IC_50_ values of 0.90 and 0.37 µg/mL, respectively. Statistical analysis showed a strong correlation between xanthone content and cholinesterase inhibition. Nonmetric multidimensional scaling analysis revealed α-mangostin and γ-mangostin of pericarp as the key metabolites contributing to cholinesterase inhibition. Due to the increasing demand of mangosteen products, repurposing of fruit waste (pericarp) has great potential for enhancement of the cognitive health of human beings.

## Introduction

*Garcinia mangostana* (mangosteen) is well known as ‘the queen of fruits’ and is one of the best tasting tropical fruits. The mangosteen fruit has dark purple or reddish pericarp, with white, soft and juicy edible aril that has slightly acidic and sweet flavour. *Garcinia mangostana* is recognised as one of the novel food in Asia, European Union, and the US due to its high antioxidant potential and traditional consumption in its countries of origin. In the US, mangosteen juice is the second best-selling herb and botanical with the total sales volume of USD 176 million and ranked 22nd for the best-selling supplement^1^.

Phytochemical analysis has been conducted to analyse chemical constituents of *G. mangostana* (mainly xanthones) using the conventional HPLC-UV^2^ and TLC^3^ methods. Although these methods have been routinely used for the analysis of xanthones, they are relatively insensitive. A HPLC-DAD-MS method has been developed to quantify seven xanthones in the pericarp, aril segments and the functional beverage^4^ and six xanthones have been identified and quantified by LC–ESI-MS^5^. However, there is little or no information about the content of xanthones such as α-mangostin in other parts of mangosteen tree. The variation in xanthone content in extracts prepared using different extraction solvents (organic or aqueous) also remained unknown. Therefore, developing more powerful analytical tools and methods for the simultaneous and systematic quantification of xanthones in different parts of *G. mangostana* is of great interest. Liquid chromatography-quadrupole of flight tandem mass spectrometric (LC-QTOF-MS) allows the generation of mass information with greater accuracy and precision and has been used to determine the molecular formula at low part per million concentrations.

Severe loss of cholinergic neurons in the nucleus basalis and associated areas that form the cholinergic forebrain area resulted in up to 90% reduction in the activities of the enzyme choline acetyltransferase, which is needed for the synthesis of the neurotransmitter acetylcholine^6^^,^^7^. It is evident that acetylcholine, a neurotransmitter essential for processing memory and learning, is decreased in both concentration and function in patients with Alzheimer’s disease as a result of reduction in its synthesis and rapid breakdown by cholinesterase enzymes^8^. Current available strategy for the treatment of Alzheimer's disease relies on blocking the breakdown of acetylcholine through cholinesterase inhibitors to improve brain functions, and possibly slow deteriorations of cognitive functions^9^. Excellent candidates from natural products are shown to improve cognitive function including *Gingko biloba* leaves extract, huperzine from *Huperzia serrata*, green tea, ginger and caffeine^10–13^, whereby some of them work by cholinesterase inhibition.

Our previous study showed that the methanol extract of *G. mangostana* pericarp and its six xanthones constituents possessed potent cholinesterase inhibitory activities with IC_50_ value in the range of 1.28–8.0 µg/mL, whereby garcinone C, γ-mangostin and α-mangostin were the most potent inhibitors among the tested xanthones^14^. Over the course of our continuing study to explore the potential cognitive enhancement properties of this plant, we aimed to compare the xanthone content in different parts of the plant and its correlation with their bioactivities. Herein, we report the analysis of six bioactive xanthones in aqueous and organic extracts of different parts of *G. mangostana* using LC-QTOF-MS and evaluate their cholinesterase inhibitory activities for the first time, and correlate the influence of xanthone content on cholinesterase inhibitory potential.

## Materials and methods

### Chemicals and reagents

Acetylthiocholine iodide (ATCl), acetylcholinesterase from *Electrophorus electricus* (electric eel) (AChE), bovine serum albumin (BSA), 5,5′-dithiobis [2-nitrobenzoic acid] (DTNB), butyrylcholinesterase from equine serum (BChE), S-butyrylthiocholine chloride and galantamine were purchased from Sigma Chemicals (St. Louis, MO, USA). HPLC grade methanol was purchased from Merck (Darmstadt, Germany). Formic acid was purchased from R & M Chemicals (United Kingdom). Marker compounds: 8-deoxygartanin (97.5% purity) was purchased from Chromadex (Irvine, CA, USA) while γ-mangostin (98.37% purity) and garcinone C (>98% purity) were obtained from Chengdu Biopurify (Chengdu, China). Mangostanol (>95% purity), 3-isomangostin (>90% purity) and α-mangostin (>95% purity) were isolated in-house following procedures described previously^14^.

### Plant materials

The raw materials of *G. mangostana* were obtained from Penang, Malaysia. The plant materials were obtained from a single tree of about 5 years old and 3m height. The green mature leaves, bark, stem and whole ripe purplish fruit were collected for this study. A voucher specimen (No. 11247) has been deposited at the herbarium, School of Biological Sciences, Universiti Sains Malaysia, Malaysia. The plant materials were separated into the following parts: leaves, bark, stem and whole fruit (the whole fruit was further separated into pericarp, aril, calyx, and stalk).

### Preparation of organic and aqueous extracts of different parts of *G. mangostana*

The different parts of *G. mangostana* were extracted with methanol or distilled water by using the maceration method. In brief, the powdered samples (20 g) were extracted with methanol or distilled water for 3 days at 60°C at raw material to solvent ratio of 1:10 (w/v). Fresh solvents were replenished every day and the resulting extracts were filtered through filter paper. The pooled extracts were evaporated under vacuum and lyophilised. The yields of the lyophilised extracts were between 0.8% and 38.2%.

### LC-QTOF-MS analyses

#### Preparation of the marker compounds for optimisation of MS

For optimisation of MS conditions, stock solutions of the marker compounds at 1000 µg/mL were prepared by accurately weighing each marker compound, then dissolving them in HPLC grade methanol and storing at 4 °C prior to analysis. Working solutions were prepared by diluting an aliquot of the stock solution with methanol to the desired range of concentrations. All the diluted solutions were filtered through a 0.22 µm PTFE syringe filter (Whatman, UK) prior to injection into the LC system.

#### LC-QTOF-MS

The LC-QTOF-MS system consisted of Ultimate 3000 RSLC (Dionex; Sunnyvale, CA) coupled to micrOTOF-QII quadrupole time-of-flight mass spectrometer in positive electrospray mode (Bruker; Billerica, MA). A Zorbax SB-C18 (150 mm X 2.1 mm, 3.5 µm) column was used for the separation of compounds of interest. The column temperature was maintained at 25 °C. Mobile phase consisted of 0.1% formic acid in ultra-high quality water (A) and 0.1% formic acid in methanol (B) at a constant flow rate of 0.2 ml/min. Gradient programme was as follows: 0–6 min 40% A, 6–15 min 10% A, 15.1–18 min 0% A, 18.1–23 min 40% A. Optimised mass spectrometer conditions were: capillary voltage 4500 V; nebuliser pressure 5 bar; dry gas 6 L/min; gas temperature 300 °C; collision energy isomangostin and α-mangostin (15 V), garcinone C (30 V), crystal violet 372 (40 V), 8-deoxygartanin (11 V), γ-mangostin (12 V), mangostanol (20 V).

#### Calibration curves, linearity ranges, limits of detection (LODs) and limits of quantification (LOQs)

The linear dynamic ranges of the LC-QTOF-MS method for the determination of the six xanthones were evaluated from a set of five solutions, at concentrations ranging from 0.025 to 50 µg/mL (some of the xanthones had much lower linear range, refer to Table 1). Each individual marker compound at a fixed concentration was consecutively injected three times. The calibration curves were constructed by plotting peak height against the analyte concentrations and the linearity of six compounds were evaluated by linear regression analysis. The LODs of the xanthones were in the range of 5 to 500 ng/mL, while their LOQs were in the range of 25 to 5000 ng/mL.

**Table 1.** Linearity, LOD and LOQ of the xanthones of *Garcinia mangostana.*

Compound	Regression equation	Linear range (ng/mL)	*R*^2^	LOD (ng/mL)	LOQ (ng/mL)
Mangostanol (1)	*y* = 0.176164*x* ± 0.045660	1000–10000	0.9998	500	1000
3-Isomangostin (2)	*y* = 1.000952*x* ± 0.002474	25–1000	0.9999	5	25
Garcinone C (3)	*y* = 0.035076*x* ± 0.030565	5000–50000	0.9987	120	5000
γ-Mangostin (4)	*y* = 0.389182*x* ± 0.000753	1000–5000	0.9910	90	1000
8-Deoxygartanin (5)	*y* = 0.595092*x* ± 0.003890	25–500	0.9969	10	25
α-Mangostin (6)	*y* = 0.480042*x* ± 0.151938	1000–10000	0.9992	50	1000

#### Quantification of xanthones by LC-QTOF-MS

Volumes of 5 µL of the extracts were injected into the system. The concentration and profile of xanthones in the extracts were determined using optimised LC-QTOF-MS parameters. Peak identifications were made by matching the retention times and the fragmentation pattern with those of the marker compounds and were quantified using the internal standard method.

### Cholinesterase inhibition assay

Cholinesterase inhibitory potential of the extracts of different parts of *G. mangostana* were determined by the spectrophotometric method as described previously^15^. For both AChE and BChE inhibitory assay, 140 µL of 0.1 M sodium phosphate buffer (pH8) was added to a 96 wells microplate followed by 20 µL of test samples and 20 µL of 0.09 units/mL of AChE or BChE enzyme. Then, 10 µL of 10 mM 5,5′-dithiobis (2-nitrobenzoic acid) (DTNB) was added into each well followed by 10 µL of 14 mM of acetylthiocholine iodide or S-butyrylthiocholine chloride. The enzymatic hydrolysis reaction as indicated by the absorbance was measured for 30 min using Tecan Infinite 200 Pro Microplate Spectrometer at 412 nm. Galantamine was used as a positive control for both assays. Absorbencies of the test samples were corrected by subtracting the absorbencies of their respective blank.

Percentage inhibition was calculated using the following formula:

$$\mathrm{Percentage}\;\mathrm{inhibition}=\frac{\text{(Absorbance of control}-\text{Absorbance of extract) }\times \;100\%}{\text{Absorbance of control}}$$

### Statistical analysis

Nonmetric multidimensional scaling (NMDS) was used to project the pairwise differences in the detected metabolites between samples. The method was selected over principle component analysis due to the presence of missing data (concentration below detection limits). A bubble plot was superimposed on the NMDS to illustrate the relative concentration of the metabolites across samples. NMDS and the corresponding bubble plots were constructed using the PRIMER7 programme package (PRIMER-E, UK). The differences in IC_50_ across plant part clusters were presented using the box plot. Significant differences between the mean values were inferred based on analysis of variance (ANOVA). Subsequent *post-hoc* analysis was conducted using Dunnett’s Test. Relationship between the xanthone content and IC_50_ of AChE and BChE was tested using Pearson’s correlation. The relationship was illustrated using a scatter plot. Both box plot and scatter plot were generated using ggplot2 package implemented under the R programme.

## Results and discussion

### Optimisation of chromatographic method

**Figure 1.**
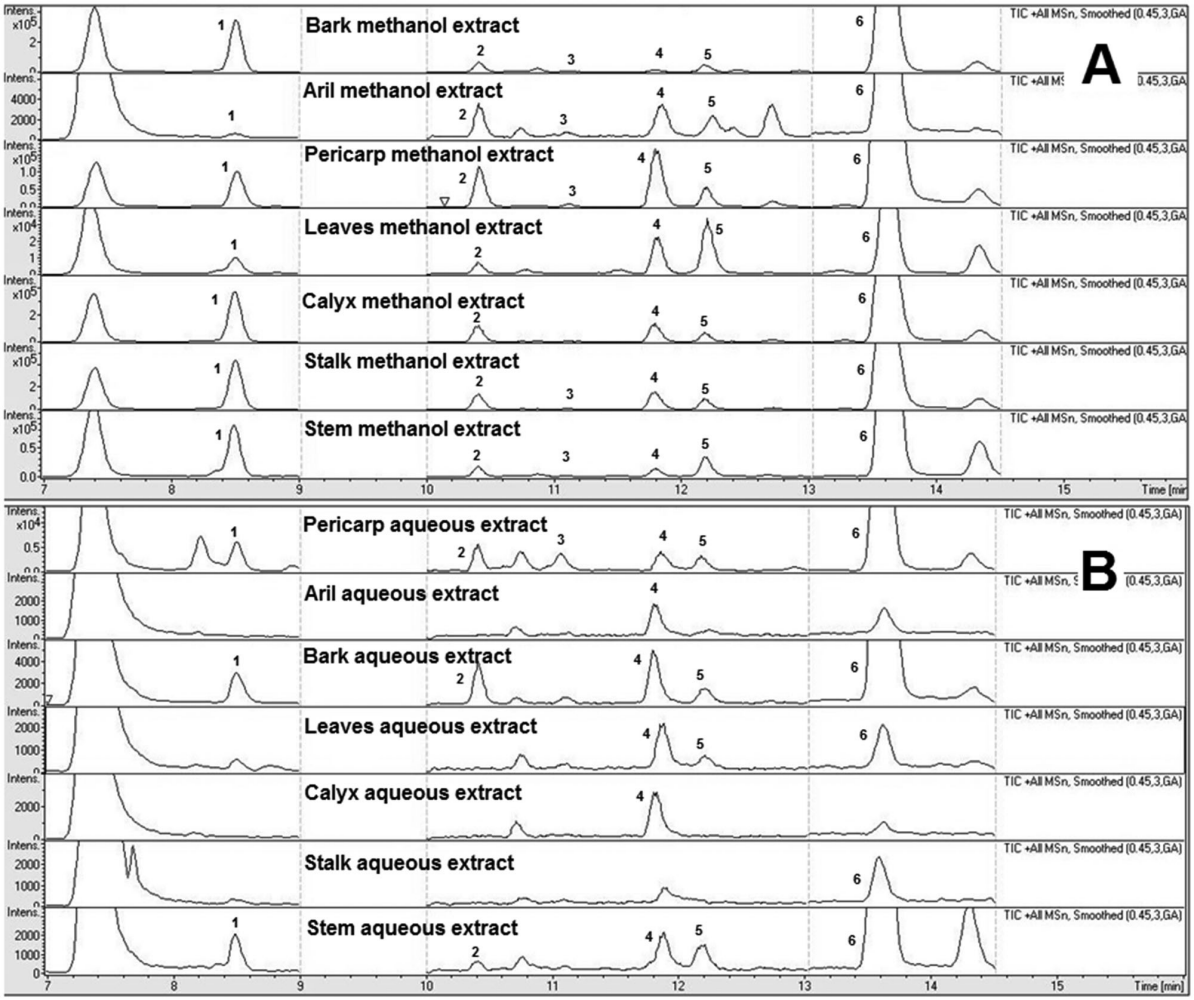
Representative total ion chromatogram of the methanol extracts (A) and aqueous extracts (B) of different parts of *Garcinia mangostana*. (1) Mangostanol ; (2) 3-Isomangostin ; (3) Garcinone C ; (4) γ-Mangostin ; (5) 8-Deoxygartanin ; (6) α-Mangostin.

For the LC-QTOF-MS method optimisation, various chromatographic parameters including mobile phase compositions, analytical columns and elution programmes were systematically optimised to acquire a better peak resolution and capacity for identification and quantification of xanthones between their adjacent peaks in the chromatogram. A small amount of formic acid was introduced to increase the peak shape and as an ionic modifier to enhance the mass response of the marker compounds in the *G. mangostana* extracts. Different reverse phase (RP) columns were used for optimisation, and Zorbax SB-C18 column was chosen due to its superiorities at satisfactory separations for all investigated analytes, acceptable peak shape, and low system back-pressure. A gradient elution programme was optimised to obtain good baseline separation for all marker compounds. Representative total ion chromatograms for the methanol and aqueous extracts of different parts of *G. mangostana* are shown in Figure 1.

### Ionisation pattern of xanthones in ESI

**Figure 2.**
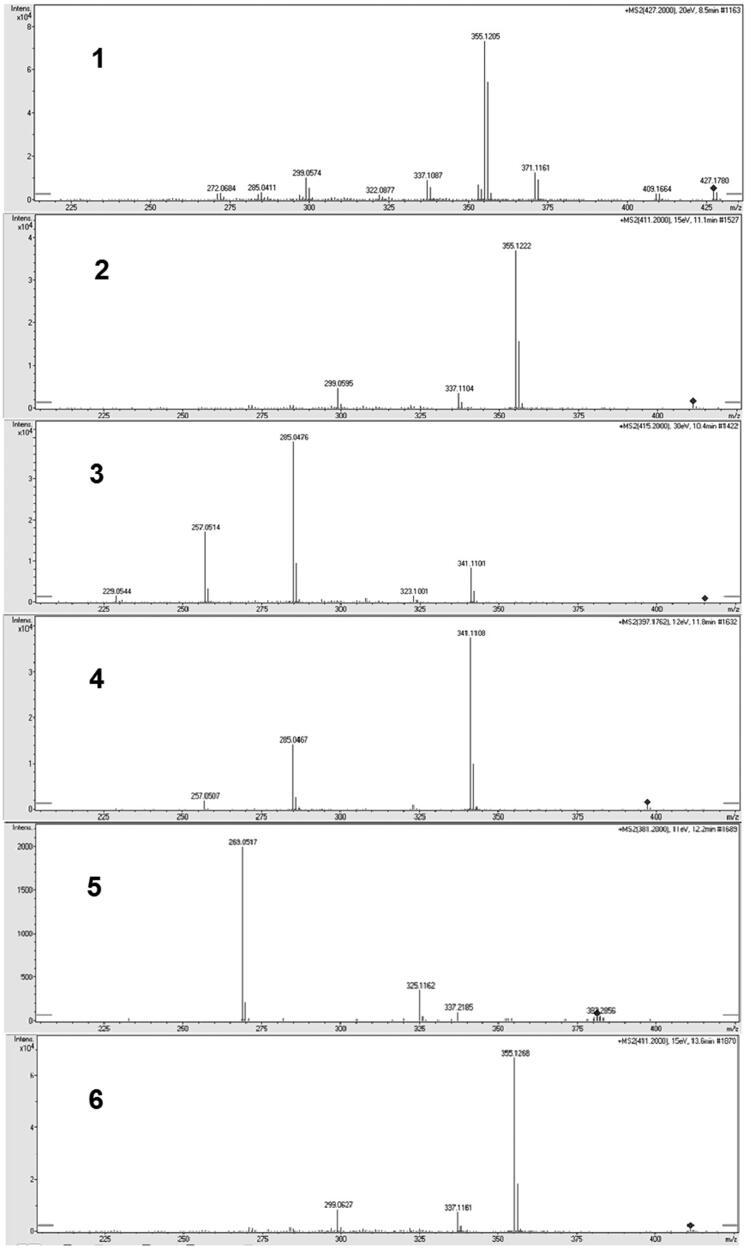
Fragmentation patterns of the xanthones. (1) Mangostanol, (2) 3-Isomangostin, (3) Garcinone C, (4) γ-Mangostin, (5) 8-Deoxygartanin, (6) α-Mangostin.

Both positive and negative ionisation modes are acceptable for the analysis of xanthones. Several groups have reported the analyses of xanthones by LC-MS in positive ionisation mode. Li et al. has identified the bioactive prenylated xanthones that inhibited the growth of *Ralstonia solanacaerum* using LC-MS in positive mode^16^. Likewise, mangiferin, a xanthone derivative was analysed and quantified in *Swertia chirayita* methanolic extract using LC-ESI/MS in positive mode^17^. In contrast, there are a number of studies that analysed and quantified prenylated xanthones in *G. mangostana* in negative ionisation mode MS^4^^,^^18^. To obtain structural information, MS/MS studies of the molecular ion of each compound were performed. All the compounds were characterised by the interpretation of their exact molecular weight, molecular formula, and characteristic MS/MS fragment ions data acquired from the Q-TOF-MS (Figure 2). The diagnostic fragmentation patterns of different xanthones are tabulated on the basis of Q-TOF-MS data of components in *G. mangostana* and six authentic marker compounds. The fragmentation pathways of the xanthones derived from *G. mangostana* were previously discussed^4^.

### Method validation and xanthones quantification

As shown in Table 1, all xanthones demonstrated good linearity (*r*^2^ > 0.99) over a range of concentrations used. The LODs and LOQs of xanthones were in the range of 5–500 ng/mL and 25–5000 ng/mL, respectively. The xanthone content in methanol and aqueous extracts of different parts of *G. mangostana* are summarised in Table 2. The highest total xanthone content was found in the methanol extract of pericarp (521.2 mg xanthones/g of extract or 185.5 mg of xanthones/g of dry powdered raw material). The total xanthone content in the methanol extracts of different parts of the plant were in the following descending order: pericarp > calyx > bark > stalk > stem > leaves > aril. Interestingly, the methanol extract of aril had 100 folds lower in total xanthone content compared to pericarp. A previous study quantified xanthones in the aril and pericarp of *G. mangostana* using HPLC-DAD-MS^4^. The authors reported 8.6 mg and 0.43 mg of γ-mangostin, 1.4 mg and 0.26 mg of 8-deoxygartanin and 33.2 mg and 2.13 mg of α-mangostin in each gram of pericarp and aril dried materials, respectively. The levels of the three xanthones in pericarp dry material were lower; however, their levels in aril were higher compared to our present study. The difference could be due to the difference in the ripening stages of the fruits and the extraction solvent used, which was methylene chloride. On the other hand, a more recent study on the xanthone analysis of six different maturity levels of mangosteen rind extract using LC-MS/MS analysis reported the γ-mangostin levels in the range of 0.94–66.13 mg per gram of extract, which were comparable with the present study (57.0 mg/g extract)^19^. In contrast to the methanol extracts, total xanthone content in the aqueous extracts are relatively much lower attributed to the relatively non-polar nature of the prenylated xanthones. The total xanthone content for aqueous extracts was in the range 2.9–7.3 mg/g of extract. Aqueous extract of bark had the highest total xanthone content followed by pericarp, stem and leaves. In addition, calyx, aril and stalk had almost similar total xanthone content.

**Table 2.** Xanthone content in the methanol and aqueous extracts of different parts of *Garcinia mangostana.*

Sample			Content of bioactive xanthones (*n* = 3) (mg/g)													Relative distribution^c^					
	Yield (%)	Mangostanol (1)		3-Isomangostin(2)		Garcinone C (3)		γ-Mangostin (4)		8-Deoxygartanin (5)		α -mangostin (6)		Total		1	2	3	4	5	6
		Extract^a^	RW^b^	Extract^a^	RW^b^	Extract^a^	RW^b^	Extract^a^	RW^b^	Extract^a^	RW^b^	Extract^a^	RW^b^	Extract^a^	RW^b^						
*Methanol extract*																					
Pericarp	35.6	8.6 ± 0.4	3.1	10.4 ± 0.6	3.7	0.5 ± 0.1	0.2	57.0 ± 12.7	20.2	11.5 ± 0.6	4.1	403.9 ± 13.1	143.7	521.2	185.5	0.8	1	0.06	5.4	1.1	38.6
Calyx	38.2	35.3 ± 2.6	13.5	7.7 ± 0.3	2.9	0.6 ± 0.0	0.2	37.2 ± 1.9	14.2	13.2 ± 1.2	5.0	221.0 ± 11.2	84.0	311.5	118.9	4.5	1	0.08	4.7	1.7	28.5
Aril	10.8	ND	ND	3.4 ± 0.2	0.4	0.004 ± 0.0	0.4 (μg/g)	0.1 ± 0.1	0.01	0.1 ± 0.0	0.01	1.2 ± 0.1	0.13	5.4	0.6	ND	1	ND	0.04	0.03	0.4
Stalk	21.7	46.9 ± 1.3	10.2	11.5 ± 0.3	2.5	1.0 ± 0.0	0.2	3.7 ± 0.1	0.8	19.5 ± 0.4	4.2	56.9 ± 1.7	12.3	73.0	15.8	4.0	1	0.09	0.3	1.6	4.9
Leaves	19.5	0.8 ± 0.1	0.2	3.9 ± 0.1	0.7	0.2 ± 0.0	0.04	3.7±.0.3	0.7	5.4 ± 0.2	1.1	2.4 ± 0.2	0.5	16.6	3.2	0.2	1	0.04	0.9	1.4	0.6
Bark	7.5	5.0 ± 0.5	0.4	10.0 ± 0.7	0.8	0.1 ± 0.0	7.5 (μg/g)	2.7 ± 0.6	0.2	15.6 ± 0.6	1.2	209.7 ± 16.9	15.7	242.3	18.2	0.5	1	0.01	0.3	1.5	20.9
Stem	14.8	8.7 ± 0.1	1.3	6.8 ± 0.3	1.0	0..1 ± 0.0	0.02	2.1 ± 0.01	0.3	6.1 ± 0.4	0.9	47.6 ± 5.4	7.0	71.5	10.6	1.2	1	0.01	0.3	0.9	6.9
*Aqueous extract*																					
Pericarp	8.6	0.06 ± 0.02	5.2 (μg/g)	3.6 ± 0.1	0.3	0.1 ± 0.0	8.6 (μg/g)	0.4 ± 0.04	0.03	0.1 ± 0.0	8.6 (μg/g)	2.5 ± 0.4	0.2	6.5	0.6	ND	1	0.02	0.1	0.03	0.6
Calyx	18.2	ND	ND	2.9 ± 0.1	0.5	ND	ND	ND	ND	0.004 ± 0.0	0.73 (μg/g)	ND	ND	2.9	0.5	ND	1	ND	ND	ND	ND
Aril	0.8	ND	ND	2.9 ± 0.0	0.02	ND	ND	ND	ND	0.01 ± 0.0	0.08 (μg/g)	ND	ND	2.9	0.002	ND	1	ND	ND	ND	ND
Stalk	13.7	ND	ND	2.9 ± 0.1	0.4	ND	ND	ND	ND	0.01 ± 0.0	1.37 (μg/g)	ND	ND	2.9	0.4	ND	1	ND	ND	ND	ND
Leaves	1.8	ND	ND	2.9 ± 0.0	0.05	ND	ND	1.4 ± 0.0	0.03	0.04 ± 0.0	0.72 (μg/g)	ND	ND	4.3	0.08	ND	1	ND	0.4	0.02	ND
Bark	3.8	ND	ND	5.3 ± 0.2	0.2	ND	ND	0.01 ± 0.0	0.38 (μg/g)	0.2 ± 0.0	7.6 (μg/g)	1.7 ± 0.1	0.06	7.3	0.3	ND	1	ND	ND	0.04	0.3
Stem	1.9	ND	ND	3.2 ± 0.1	0.1	ND	ND	0.03 ± 0.0	0.57 (μg/g)	0.2 ± 0.1	3.8 (μg/g)	0.9 ± 0.1	0.02	4.8	0.009	ND	1	ND	0.01	0.05	0.3

ND: Not determined because the level was below quantification limit. Numbers label: (1) mangostanol; (2) 3-isomangostin; (3) garcinone C; (4) γ-mangostin; (5) 8-deoxygartanin; (6) α-mangostin.

^a^‘Extract’ means the content of the compounds in the extract, e.g., 8.6 mg of mangostanol is present in each g of methanol extract of pericarp.

^b^'RW’ means the content of the compounds in the dry powdered raw material, e.g., 3.1 mg of mangostanol is present in each g of dry powdered pericarp raw material.

^c^The relative distribution indicates the ratio of the compounds relative to 3-isomangostin. The idea of relative distribution is to indicate the magnitude of the ratio between two compounds, e.g., in methanol extract of pericarp, there is 38.6 times more α-mangostin, 5.4 times more γ-mangostin and 1.1 times more 8-deoxygartanin than 3-isomangostin.

The relative distribution of the xanthones in the methanol extracts showed that α-mangostin was abundant in the pericarp (403.9 mg/g extract), calyx (221.0 mg/g extract), bark (209.7 mg/g extract) and stalk (56.9 mg/g extract). On the other hand, 8-deoxygartanin was abundant in the stalk (19.5 mg/g extract) and leaves (5.4 mg/g extract), whereas, 3-isomangostin was abundant in the aril (3.4 mg/g extract). The distribution of garcinone C was relatively smaller compared with other xanthones. On the contrary, the relative distribution of the xanthones in aqueous extract showed that 3-isomangostin was abundant among the xanthones in all parts of the plant.

### Cholinesterase inhibitory activities of the extracts

Cholinesterase inhibitor has been widely accepted as one of the effective strategies for symptomatic treatment of AD^20^^,^^21^. As the disease progress, the activities of AChE declines in certain brain regions to 10–15% of normal activities, whereas BChE activities rise to partially compensate for the loss in AChE activities. Current available drugs for the treatment of AD predominantly are AChE inhibitors. Some investigations showed that BChE might possess as an interesting target for the treatment of AD^22^.

In this study, the cholinesterase inhibitory activities of the *G. mangostana* extracts were evaluated. The cholinesterase inhibitory activities of the methanol extracts of different parts of the plant are compared with the aqueous extracts and summarised in Table 3. The methanol and aqueous extracts were initially screened for their cholinesterase inhibitory activities at 100 µg/mL. Extracts that had more than 50% inhibition at 100 µg/mL were further evaluated for their IC_50_. The IC_50_ values were in the range of 0.37–72.22 μg/mL for both AChE and BChE. The methanol extracts of the pericarp and calyx showed the most potent inhibitory activities against AChE and BChE enzymes, with IC_50_ values of 0.90 μg/mL and 0.37 μg/mL, respectively. The methanol extract of calyx was a BChE selective inhibitor with a selectivity index of 26 while the methanol extracts of pericarp and bark are AChE selective inhibitor with a selectivity index of more than 2.

**Table 3.** Cholinesterase inhibitory activities of the methanol and aqueous extracts of different parts of *Garcinia mangostana.*

Sample	% of inhibition at 100 µg/mL		IC_50_ (µg/mL)		Selectivity	
	AChE	BCHE	AChE	BChE	AChE	BChE
*Methanol extract*						
Pericarp	82.19 ± 2.89	74.63 ± 1.03	0.90 ± 0.10	1.94 ± 0.14	2.15	0.46
Calyx	92.92 ± 3.86	94.10 ± 4.12	9.71 ± 1.20	0.37 ± 0.04	0.04	26.24
Aril	22.00 ± 0.48	5.26 ± 0.37	ND	ND	ND	ND
Stalk	65.54 ± 3.51	80.11 ± 0.74	22.81 ± 0.72	20.84 ± 0.02	0.91	1.09
Leaves	32.76 ± 2.09	76.46 ± 1.67	ND	25.83 ± 0.77	ND	ND
Bark	83.90 ± 1.35	76.46 ± 0.18	7.88 ± 0.82	19.89 ± 2.68	2.52	0.39
Stem	94.0 3 ± 1.75	89.95 ± 0.78	22.64 ± 0.77	1.82 ± 0.16	0.08	12.44
*Aqueous extract*						
Pericarp	55.37 ± 1.53	82.59 ± 0.09	52.74 ± 8.98	11.14 ± 0.61	0.21	4.73
Calyx	17.90 ± 3.13	NI	ND	ND	ND	ND
Aril	NI	NI	ND	ND	ND	ND
Stalk	59.20 ± 3.60	2.36 ± 0.37	72.22 ± 2.58	ND	ND	ND
Leaves	15.70 ± 0.93	65.19 ± 0.56	ND	38.81 ± 1.84	ND	ND
Bark	NI	15.09 ± 8.56	ND	ND	ND	ND
Stem	11.60 ± 1.16	11.23 ± 0.93	ND	ND	ND	ND
Galantamine			0.27 ± 0.07	5.55 ± 0.24	20.55	0.04

Each value represents mean ± SD of triplicates. NI: No inhibition at 100 µg/mL. ND: Not determined.

Selectivity against AChE: IC_50_BChE/IC5_0_AChE.

Selectivity against BChE: IC_50_AChE/IC5_0_BChE.

In contrast to the methanol extract, the aqueous extract displayed moderate to poor inhibitory activities against both AChE and BChE. Aqueous extracts of the pericarp and stalk showed moderate inhibitory potency towards AChE, while aqueous extracts of the pericarp and leaves extract demonstrated moderate inhibitory activities against BChE. Our results show that the preicarp extracts from two different extraction solvents exhibit inhibitory activities against both AChE and BChE. In line with the vast differences in cholinesterase inhibitory activities between the extracts from two extraction solvent systems, further analyses were carried out to determine the relationship between xanthone content with the cholinesterase inhibitory activities.

### Correlation between xanthone content and cholinesterase inhibitory activities

**Figure 3.**
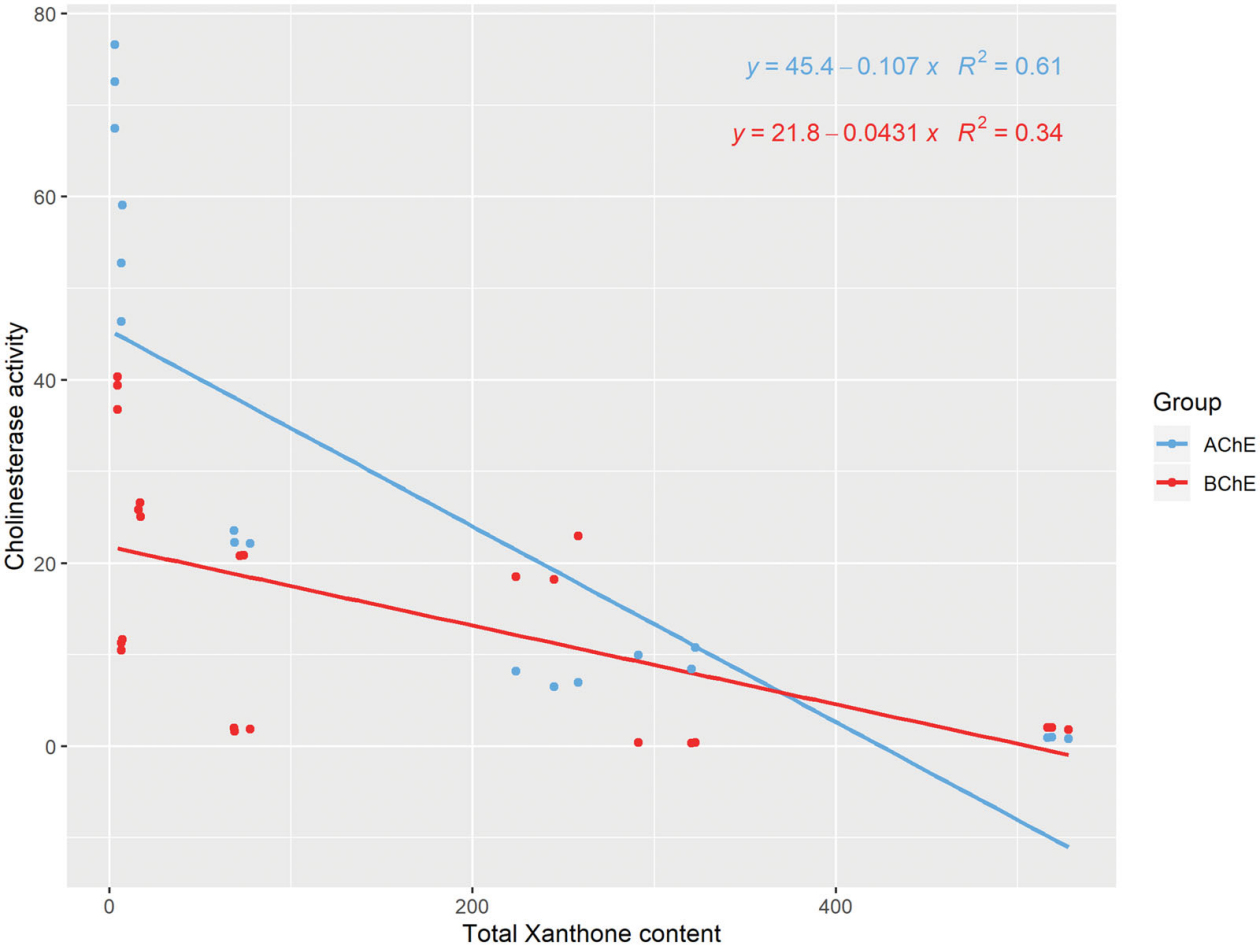
Scatter plot of IC_50_ of AChE and BChE versus total xanthone content.

In the present study, we sought to delineate xanthone content in the different parts of *G. mangostana* with their cholinesterase activities. Pearson’s correlation analysis revealed that the total xanthone content was significantly correlated with IC_50_ for AChE (*R* = −0.780, *p* < 0.01) and BChE (*R* = −0.585, *p* = 0.003). Specifically, a stronger correlation was detected between xanthone content and IC_50_ of AChE than IC_50_ of BChE (Figure 3).

**Figure 4.**
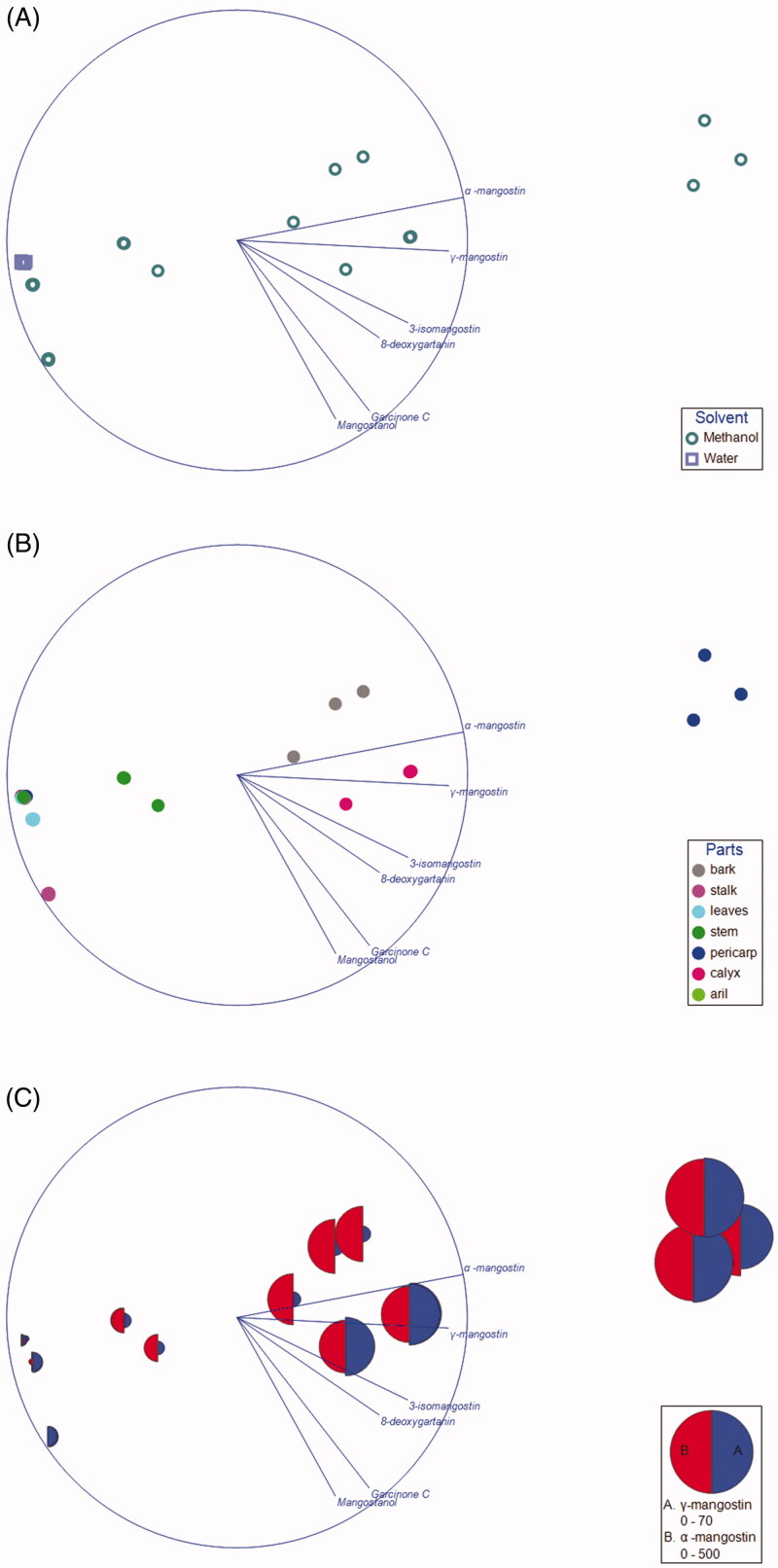
Nonmetric Multidimensional Scaling Ordination of the xanthones. The loading of the variables was projected on the ordination plot. The sample was labelled based on (A) solvent, (B) plant parts. (C) Bubble plot based on the concentration of α-mangostin and γ-mangostin superimposed on the NMDS.

**Figure 5.**
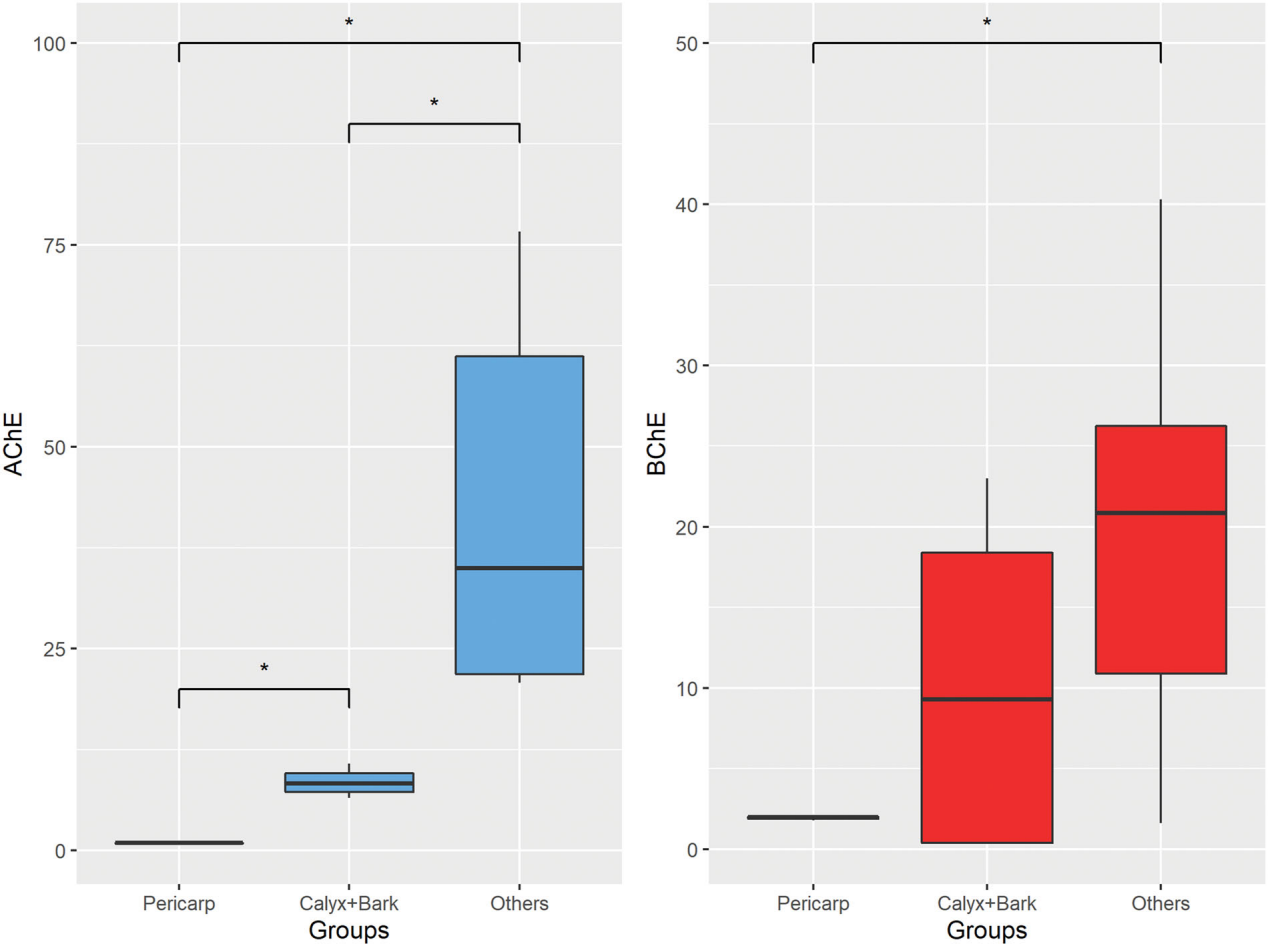
Box plot for comparison of IC_50_ between “Pericarp”, “Bark + Calyx” and “Others”. (A) AChE, (B) BChE.

A total of six xanthones, namely α-mangostin, γ-mangostin, 3-isomangostin, 8-deoxygartanin, garcinone C and mangostanol, were detected in the aqueous and/or methanol extracts. Based on the NMDS ordination (Figure 4(A)), the composition of these metabolites in the methanol extracts showed greater variation than the aqueous extract. Three main clusters consisted of (1) pericarp, (2) bark and calyx (3) others were apparent when the samples were labelled based on plant parts (Figure 4(B)). Among the metabolites, α-mangostin and γ-mangostin showed the strongest contribution to the separation along the horizontal axis. Indeed, the bubble plot superimposed on the NMDS showed highest level of α-mangostin and γ-mangostin in pericarp, while the lowest level of both in others (Figure 4(C)). Despite the fact that garcinone C was the most potent AChE inhibitor among the xanthones as reported in our previous study^14^, the present findings suggest that α-mangostin and γ-mangostin are the key metabolites contributing to the cholinesterase inhibition. For the cholinesterase inhibition, only the methanol extracts of different parts of *G. mangostana* showed appreciable activities. Based on the activities, the different parts of the plant can be clustered into three groups based on their IC_50_ values (refer to the box plot in Figure 5). Further statistical analysis revealed a significant difference for the IC_50_ of AChE between all three clusters (“Pericarp”, “Bark + Calyx”, and “Others”), suggesting that the AChE inhibitory activities of the pericarp is significantly superior to any other parts of the plant. Conversely, that is not the case for BChE inhibition as can be seen by the overlapping between the three clusters. Significant difference in IC_50_ for BChE was only achieved between “Pericarp” and “Others”.

Our previous paper describes on bioactivity-guided isolation of potential cholinesterase inhibitors from *G. mangostana* pericarp. We identified six bioactive xanthones, namely α-mangostin, γ-mangostin, mangostanol, 3-isomangostin, garcinone C and 8-deoxygartanin, of which the most potent inhibitor of AChE was garcinone C while γ-mangostin was the most potent inhibitor of BChE^14^. In the present study, we determined the content of the bioactive xanthones in different parts of the plant to identify the potential source of the bioactive xanthones. In addition, we tested their cholinesterase inhibition to validate our hypothesis that xanthones are the chemical constituents responsible for the cholinesterase inhibitory activities of *G. mangostana*. The findings clearly support our hypothesis, and this is the first study to show that pericarp has the highest content of xanthones among all the other parts of *G. mangostana*.

The *G. mangostana*extracts showed comparable inhibitory activities to the well known AChE inhibitory plants such as *Huperzia*^23^ (the plant is widely used in traditional Chinese medicine to enhance memory from which huperzine A, a commercially available food supplement for the improvement of memory is obtained) and *Gingko biloba*^24^ (commonly used to improve memory). It is worth to mention that the methanol extract of *G. mangostana* pericarp showed more potent AChE inhibition (IC_50_ of 0.90 µg/mL) compared to *Huperzia serrata*^23^ (IC_50_ of 12.23 µg/mL) and *G. biloba*^24^ (IC_50_ of 252.1 µg/mL). In addition, *G. mangostana* showed better AChE inhibition than the Ayurvedic medicinal plants used for cognitive disorders^25^, such as *Bacopa monniera* (IC_50_ of 523 µg/mL), *Centella asiatica* (IC_50_ of 890 µg/mL), *Emblica officinalis* (IC_50_ of 53.5 µg/mL), *Glycyrrhiza glabra* (IC_50_ of 418 µg/mL), *Tinospora cordifolia* (IC_50_ of 230 µg/mL) and *Withania somnifera* (IC_50_ of 124 µg/mL).

## Conclusion

In conclusion, methanol extract of the pericarp contained the highest total xanthones among the organic and aqueous extracts of different parts of *G. mangostana*. α-Mangostin was the major xanthone in the methanol extracts of pericarp, calyx and bark and stem, while 3-isomangostin was the major xanthone in the aqueous extracts of all parts of the plant. On cholinesterase inhibitory potential, the methanol extracts of pericarp and calyx had the most potent inhibitory activities against AChE and BChE with IC_50_ values of 0.90 and 0.37 µg/mL, respectively. The total xanthone content was found to be well correlated with their cholinesterase inhibitory activities. Since there is an increasing demand for mangosteen products, repurposing of fruit waste (pericarp) enriched with bioactive xanthones has great potential for enhancement of the cognitive health of human beings. The series of prenylated xanthones found in *G. mangostana* are attractive lead molecules in the field of medicinal chemistry for further structural modification and optimisation in the search of potent cholinesterase inhibitor with favourable pharmacokinetics and safety profiles.
